# Preparation of a Nanomaterial–Polymer Dynamic Cross-Linked Gel Composite and Its Application in Drilling Fluids

**DOI:** 10.3390/gels11080614

**Published:** 2025-08-05

**Authors:** Fei Gao, Peng Xu, Hui Zhang, Hao Wang, Xin Zhao, Xinru Li, Jiayi Zhang

**Affiliations:** 1 National Engineering Research Center for Oil & Gas Drilling and Completion Technology, School of Petroleum Engineering, Yangtze University, Wuhan 430100, China; 2 Hubei Key Laboratory of Oil and Gas Drilling and Production Engineering, Yangtze University, Wuhan 430100, China; 3 State Key Laboratory of Low Carbon Catalysis and Carbon Dioxide Utilization, Yangtze University, Wuhan 430100, China; 4 Sinopec North China Company, Zhengzhou 450006, China; 5 No. 9 Oil Production Plant of PetroChina Changqing Oilfield Company, Yinchuan 750001, China; 6 No. 11 Oil Production Plant of PetroChina Changqing Oilfield Company, Yinchuan 750001, China; 7 Shale Oil Development Branch of PetroChina Changqing Oilfield Company, Qingyang 745100, China

**Keywords:** drilling fluid, dynamic gel, modified nano-SiO_2_, plugging, wellbore stability

## Abstract

During the process of oil and gas drilling, due to the existence of pores or micro-cracks, drilling fluid is prone to invade the formation. Under the action of hydration expansion of clay in the formation and liquid pressure, wellbore instability occurs. In order to reduce the wellbore instability caused by drilling fluid intrusion into the formation, this study proposed a method of forming a dynamic hydrogen bond cross-linked network weak gel structure with modified nano-silica and P(AM-AAC). The plugging performance of the drilling fluid and the performance of inhibiting the hydration of shale were evaluated through various experimental methods. The results show that the gel composite system (GCS) effectively optimizes the plugging performance of drilling fluid. The 1% GCS can reduce the linear expansion rate of cuttings to 14.8% and increase the recovery rate of cuttings to 96.7%, and its hydration inhibition effect is better than that of KCl and polyamines. The dynamic cross-linked network structure can significantly increase the viscosity of drilling fluid. Meanwhile, by taking advantage of the liquid-phase viscosity effect and the physical blocking effect, the loss of drilling fluid can be significantly reduced. Mechanism studies conducted using zeta potential measurement, SEM analysis, contact angle measurement and capillary force assessment have shown that modified nano-silica stabilizes the wellbore by physically blocking the nano-pores of shale and changing the wettability of the shale surface from hydrophilic to hydrophobic when the contact angle exceeds 60°, thereby reducing capillary force and surface free energy. Meanwhile, the dynamic cross-linked network can reduce the seepage of free water into the formation, thereby significantly lowering the fluid loss of the drilling fluid. This research provides new insights into improving the stability of the wellbore in drilling fluids.

## 1. Introduction

Wellbore instability is a pervasive issue in drilling operations, manifesting as wall collapse, hole shrinkage, and stuck pipe, which significantly compromise drilling efficiency and safety [1,2]. The primary cause of these problems is the hydration and dispersion of shale, particularly when exposed to water-based drilling fluids. The clay minerals within shale absorb water, leading to swelling and a subsequent decline in mechanical strength. Addressing wellbore instability necessitates the development of advanced plugging agents and inhibitors that can effectively mitigate shale hydration. However, conventional solutions often fall short in complex geological conditions due to limitations in thermal stability, dispersion capability, or singular mechanisms of action [1,3]. These limitations necessitate advanced materials capable of deeper penetration into shale microstructures while maintaining stability under harsh downhole environments.

To tackle wellbore instability, previous research has explored various chemical strategies, including the use of polymer inhibitors, inorganic salts, and nanomaterials. Polymer inhibitors function by forming a physical barrier on the shale surface to restrict water ingress, while inorganic salts reduce hydration swelling by altering the ionic strength of drilling fluids [2,3]. Although these methods have shown promise, their effectiveness is often compromised in high-temperature environments or complex geological settings [4,5].

Recent advancements in drilling fluid plugging agents have focused on combining physical and chemical interactions to enhance plugging efficiency [6,7]. For example, polyurethane-based plugging agents have demonstrated notable thermal stability and sealing capabilities but still require improvement for high-temperature applications [8]. The incorporation of nanomaterials has emerged as a promising direction, with nanoparticles capable of penetrating micro-pores and fractures in shale to form dense plugging layers [9].

Recent research has shifted toward nanomaterials—notably nano-SiO_2_—as a promising alternative owing to their high specific surface area, mechanical robustness, and ability to seal micro-fractures through physical plugging [10,11]. However, unmodified nano-SiO_2_ suffers from intrinsic hydrophilicity, leading to aggregation and poor dispersion in organic phases, which restricts its effectiveness in drilling fluids [12,13,14]. To overcome this, hydrophobic modification strategies (e.g., covalent grafting of silane groups or cationic surfactants [15,16]) have emerged to enhance colloidal stability and interfacial compatibility [15,16,17]. These modifications enable synergistic physical-chemical interactions—critical for forming durable, high-temperature-resistant sealing networks.

The hydrophobic modification of nano-SiO_2_ has evolved through surface and in situ modification methods. Surface modification involves introducing hydrophobic groups, such as trimethylchlorosilane (TMCS), to convert the surface from hydrophilic to hydrophobic [17,18]. In situ modification leverages sol–gel processes to incorporate hydrophobic groups into the nano-SiO_2_ structure [14,19]. More recently, long-chain cationic surfactants with silane groups have shown great potential [20]. These surfactants form covalent bonds with nano-SiO_2_ through silane hydrolysis and establish ionic bonds via electrostatic interactions, achieving stable anchoring on the nano-SiO_2_ surface [21,22]. This dual-action modification not only enhances the hydrophobicity of nano-SiO_2_ but also improves its stability in complex environments, offering a new pathway for developing high-performance plugging agents [23,24,25].

Despite these advances, two critical gaps persist: (1) most studies focus either on nanoparticle modification or polymer gel design, neglecting synergistic integration [26,27]; (2) dynamic gel–nanocomposite networks for multi-mechanistic wellbore stabilization remain underexplored [28,29]. To address these gaps, this study aims to develop and characterize a novel dynamically cross-linked nanocomposite (GCS) by integrating hydrophobically modified nano-SiO_2_ with a poly (AM-AA) gel matrix, explicitly targeting enhanced plugging efficiency and shale stabilization under extreme conditions.

In this study, we first modified the nano-SiO_2_ powder to improve its dispersibility. Subsequently, P(AM-AAC) polymer was prepared by free radical polymerization, and then the modified nano-silica was combined with the polymer to prepare the composite system GCS with a dynamic cross-linked gel network. The structural characteristics of GCS were analyzed by advanced characterization techniques such as Fourier transform infrared spectroscopy (FTIR), thermogravimetric analysis (TGA), and scanning electron microscopy (SEM). Through a series of experimental evaluations, the optimization effect of GCS on the sealing performance of drilling fluid has been proved. Finally, through zeta potential measurement, SEM analysis, contact angle measurement and capillary force assessment, the mechanism by which GCS stabilizes the wellbore was revealed, thereby providing new insights into wellbore stability in drilling fluids. The scientific novelty of this work lies in two key aspects: (1) dynamic gel network: hydrogen-bonded P(AM-AA) polymer networks are synergized with modified nano-SiO_2_ to create a self-reinforcing plugging structure; (2) multi-mechanistic stabilization: GCS simultaneously leverages physical pore sealing, hydrophobic barrier formation, and electrostatic shale inhibition—overcoming the singular-action limitations of existing solutions.

## 2. Results and Discussion

### 2.1. Characterization of Hybrid Nanoparticle SiO_2_@KH550 and P(AM-AAC)

#### 2.1.1. FTIR Spectra

**Figure 1:**
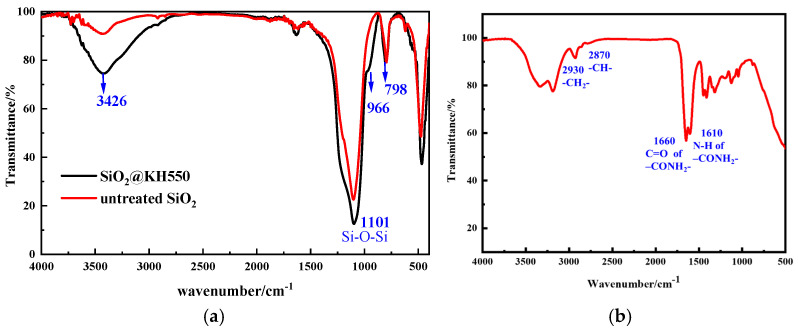
FT-IR spectra of (**a**) untreated nano-SiO_2_ and SiO_2_@KH550; (**b**) copolymer of P(AM-AAC).

SiO_2_@KH550(FNS): The structure and spectrum of the nanomaterials were analyzed by infrared spectroscopy. As shown in Figure 1a, the infrared spectrum of SiO_2_ has a strong and broad peak at 3426 cm^−1^, which is the -OH stretching vibration peak. Compared with SiO_2_@KH550, the peak intensity is weakened, indicating that the hydrophilicity of nano-SiO_2_ is reduced and the -Si-OH structural units are decreased during the modification process. After modification, a peak appears at 966 cm^−1^ in SiO_2_, indicating that KH550 has successfully modified nano-SiO_2_. The peak at 1140 cm^−1^ is that of the Si-O-Si antisymmetric vibration. The peak at 960 cm^−1^ is that of the Si-OH bending vibration, and 798 cm^−1^ is the absorption peak of the Si-O-Si symmetric stretching vibration [13,15].

P(AM-AAC): The infrared spectrum of the polymer P(AM-AAC) was tested, as shown in Figure 1b. Moderate absorption peaks at 2930 cm^−1^ and 2870 cm^−1^ are attributed to C-H asymmetric and symmetric stretching vibrations of methylene (-CH_2_^-^) and methine (-CH^-^) groups in the polymer backbone and side chains. The distinct peak at 1660 cm^−1^ corresponds to C=O stretching of the amide group (-CONH_2_). A strong absorption peak observed at 1610 cm^−1^ exhibits dual characteristics: (i) N-H bending vibration of the amide group (amide II band), (ii) C=O asymmetric stretching of carboxylate groups (-COO^−^), suggesting potential ionization or salt formation. Concomitant presence of peaks at 1408 cm^−1^ (COO^−^ symmetric stretch) and 1610 cm^−1^ collectively indicates the successful incorporation of acrylic acid units, with carboxyl groups primarily existing in ionized form (-COO^−^). In summary, FTIR confirms the formation of acrylamide–acrylic acid copolymer, evidenced by diagnostic amide (-CONH_2_) and carboxylate (-COO^−^) signatures. The spectral profile is consistent with significant intermolecular interactions within the material.

#### 2.1.2. TGA

**Figure 2:**
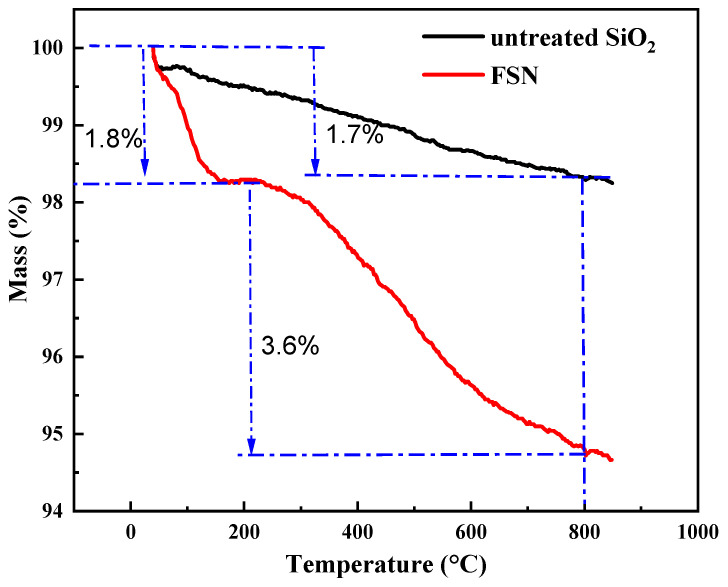
The TGA curves of untreated nano-SiO_2_ and SiO_2_@KH550.

The TGA curves of SiO_2_ and SiO_2_@KH550 are shown in Figure 2. SiO_2_ has good thermal stability, with a relatively small overall mass loss. Only 1.7% of the mass was lost for untreated SiO_2_, and this mass loss was mainly due to the evaporation of adsorbed water on the surface of SiO_2_ and the dehydration condensation of silanol groups in SiO_2_. SiO_2_@KH550 also has good thermal stability, as shown in the thermogravimetric curve in Figure 2. Before the temperature reaches 209 °C, the mass loss of SiO_2_@KH550 is small, only 1.8%, and this weight loss is mainly due to the evaporation of surface water. This is because the modifier DTPAC and SiO_2_ are bonded by both Si-O-Si chemical bonds and electrostatic forces, making the adsorbed modifier on the surface of SiO_2_ less likely to detach due to high temperature. After 209 °C, the mass loss of SiO_2_@KH550 increases significantly, with a 3.6% mass loss in the 209–800 °C stage. The mass loss in this stage is due to the partial thermal decomposition of the adsorbed modifier on the surface of SiO_2_ and the dehydration condensation of silanol groups at extremely high temperatures. However, the mass loss of FSN is only 5.4%, indicating that SiO_2_@KH550 has excellent high-temperature resistance and is suitable for high-temperature environments underground.

#### 2.1.3. Particle Dispersibility

**Figure 3:**
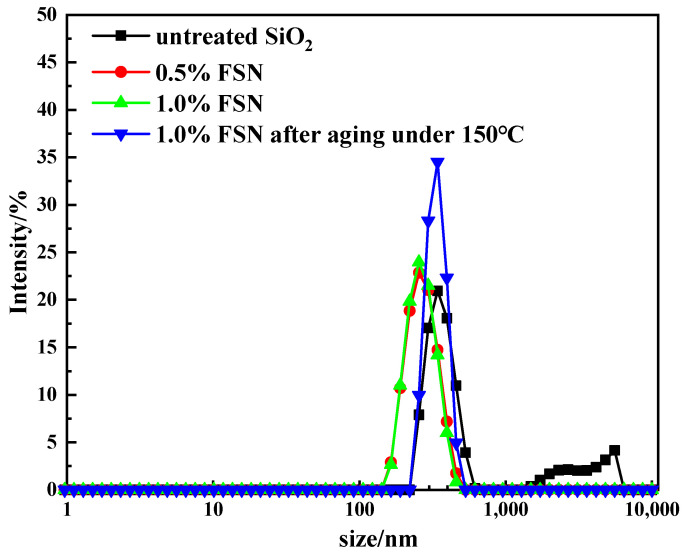
The particle size distribution of untreated SiO_2_ and FSN before and after heating at 150 °C.

Figure 3 shows the particle size distribution of untreated SiO_2_ and FSN before and after heating at 150 °C. The particle size distribution of unmodified nano-SiO_2_ exhibited a bimodal distribution, with particles existing at both nanometer and micrometer scales. This indicates poor dispersibility of the unmodified nano-SiO_2_, as the particles tend to aggregate. In contrast, the modified nano-SiO_2_ showed a narrow particle size distribution centered around 300 nm, with minimal changes in particle size observed as the concentration increased. This suggests that the modification significantly enhanced the dispersion stability of the nanoparticles.

Comparative analysis of the particle size distribution of 1% FSN dispersion before and after aging at 150 °C revealed a slight increase in particle size following thermal exposure. This indicates that high-temperature aging may lead to partial degradation of the surface groups on the FSN nanoparticles, thereby reducing their dispersibility. However, the overall change in particle size was not substantial, suggesting that FSN-modified nano-SiO_2_ maintains relatively good stability even under elevated temperature conditions.

**Figure 4:**
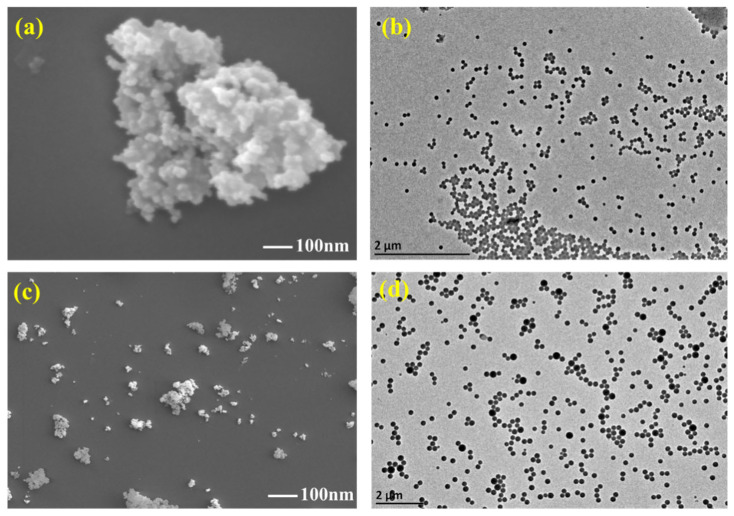
(**a**) SEM images of untreated nano-SiO_2_; (**b**) TEM images of untreated nano-SiO_2_; (**c**) SEM images of SiO_2_@KH550; (**d**) SEM images of SiO_2_@KH550.

Figure 4 shows the micromorphology of unmodified nano-silica and hydrophobic modified SiO_2_@KH550. Unmodified nano-SiO_2_ particles exhibit a high tendency to aggregate due to their small size and the presence of numerous hydroxyl groups on the surface, which endow them with an extremely high specific surface area and activation energy. As a result, these particles tend to form clusters, creating a floc-like network structure, as observed in the SEM and TEM images, shown in Figure 4a,b. The TEM images reveal that the particles are poorly dispersed, with no clear separation between individual particles, indicating significant aggregation. In contrast, hydrophobic modification significantly enhances the dispersibility of nano-SiO_2_ particles. The modified particles exhibit improved dispersion with distinct and well-separated contours, as shown in the SEM and TEM images in Figure 4c,d. This enhanced dispersion is attributed to the positive charge on the surface of the modified nano-SiO_2_ particles, which induces electrostatic repulsion between adjacent particles, thereby preventing aggregation. The TEM images clearly demonstrate the individualized particles with minimal aggregation, highlighting the effectiveness of the hydrophobic modification in improving the overall dispersibility of nano-SiO_2_.

#### 2.1.4. Preparation of the Dynamic Cross-Linked P(AM-AAC)/SiO_2_@KH550 Nanocomposite Gel

**Figure 5:**
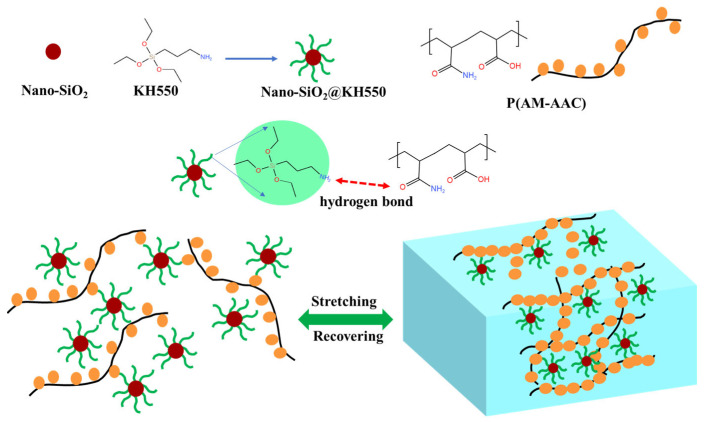
Schematic illustration of preparation of the hybrid nanoparticle SiO_2_@KH550 and the densely dynamic hydrogen bond cross-linked (P(AM-AAC)/SiO_2_@KH550 nanocomposite hydrogel.

P(AM-AAC) was polymerized with the prepared SiO_2_@KH550 as a dynamic cross-linking agent to obtain the physically cross-linked nanocomposite hydrogel P(AM-AAC)/SiO_2_@KH550, as shown in Figure 5. The typical preparation process is: Dissolve 0.05 g of SiO_2_@KH550 mixed nanoparticles in 50 mL of deionized water and ultrasonically disperse to obtain a uniform solution. Then, 15 g of AM and AAC were added to the above solution. Finally, in a nitrogen atmosphere, the initiator APS was added, and the polymerization reaction was carried out at 70 °C for 6 h. The hydrogel obtained was denoted as GCS (P(AM-AAC)/SiO_2_@KH550.

### 2.2. Plugging Performance Evaluation

#### 2.2.1. Penetration Plugging Apparatus

PPA experiments were adopted to evaluate the plugging ability of base slurry using special sand pans with pore sized of 30 μm and 90 μm, respectively. The experimental temperature was 150 °C and the pressure was 3.5 MPa [23,24].

**Figure 6:**
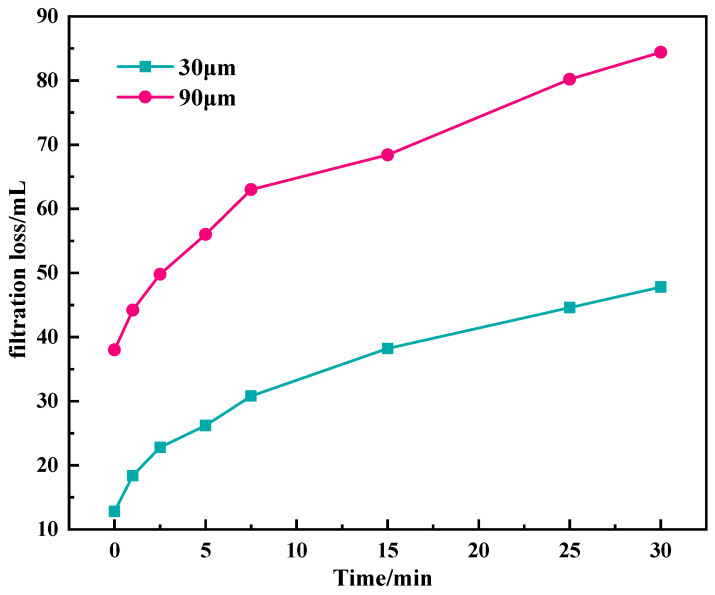
Results of penetration plugging apparatus (PPA) of base mud.

According to Figure 6, for the 30 μm sand pan, the instantaneous filtration loss is 12.8 mL, and the filtration loss reaches 47.8 mL within 30 min, showing an unsatisfactory plugging performance. For 90 μm sand pan, the instantaneous filtration loss reached 38 mL. After the formation of instantaneous mud cake, the filtration loss gradually decreased, and the filtration loss increased to 84.4 mL within 30 min. The results showed that it was difficult for base mud to effectively plug the 90 μm large pores, and the large instantaneous filtration loss was easy to cause the instantaneous intrusion of filtrate, resulting in wellbore instability.

**Figure 7:**
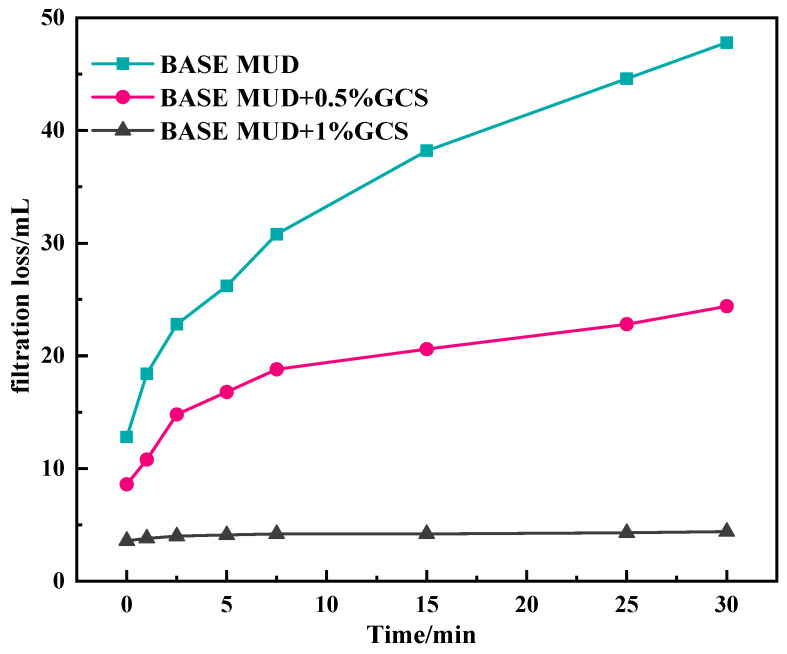
Results of penetration plugging apparatus (PPA) of base mud with FSN for 30 micro sand sheet.

According to Figure 7, for 30 μm sand sheet, the sealing effect of adding GCS is significantly better than that of the base slurry. Adding 0.5% GCS can reduce the 30 min filtration loss to 24.4 mL, and the instantaneous filtration loss is only 8.6 mL. When the GCS dosage reached 1%, the instantaneous filtration loss was reduced to 3.6 mL, and the filtration loss within 30 min was only 4.4 mL. Moreover, it can be found that with the increase in time, the curve gradually tends to be smooth, indicating that the dense sealing layer is formed on the surface of the sand disk, which effectively improves the permeability sealing performance.

**Figure 8:**
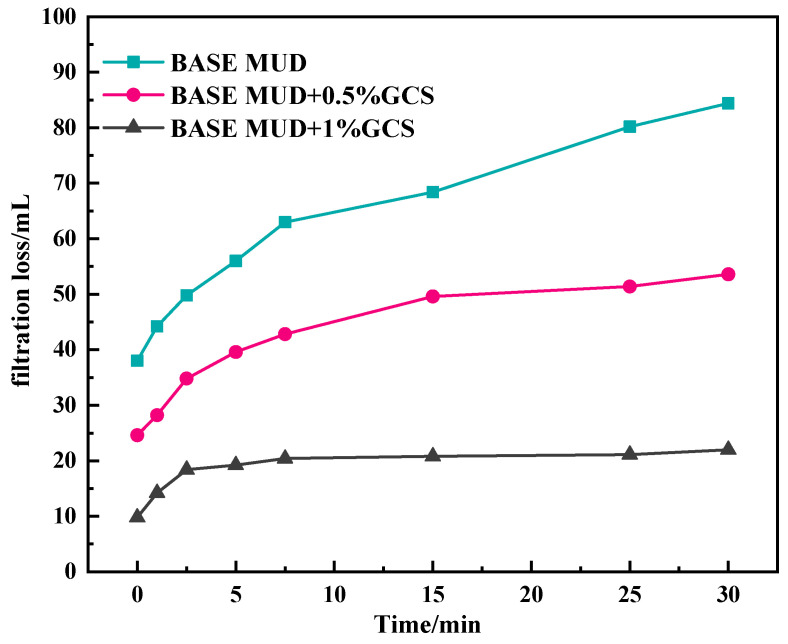
Results of penetration plugging apparatus (PPA) of base mud with FSN for 90 micro sand sheet.

As can be seen from Figure 8, for 90 μm sand pan, when 1% GCS is added, the instantaneous filtration loss drops sharply to 24.6 mL and increases to 53.4 mL within 30 min, which significantly decreases compared with the base slurry filtration loss. With the concentration of the sealing agent increasing to 1%, the instantaneous filtration loss decreased to only 9.8 mL, which indicates that the addition of the FSN can quickly form a dense sealing layer in the sand sheet with 90 micron pores, effectively reduce the intrusion of drilling fluid filtrate into the formation, and is conducive to wellbore stability. The high plugging efficiency demonstrates GCS’s capability to physically seal shale micro-fractures, directly preventing fluid invasion.

#### 2.2.2. Linear Expansion Experiment

**Figure 9:**
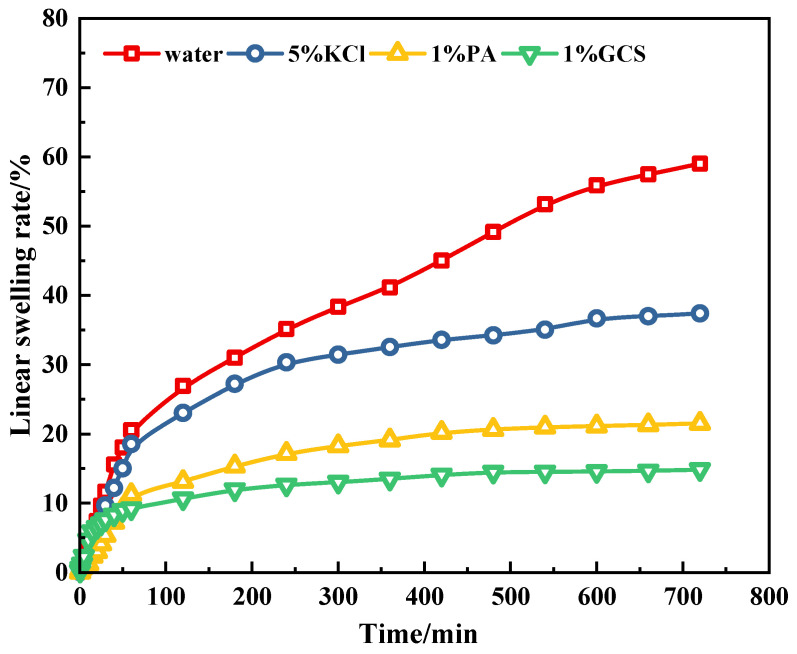
The linear expansion experiments of water, inhibitors and GCS.

Figure 9 shows the linear swelling rates of the prepared bentonite cakes in water, 5% KCl solution, 1% polyamine solution, and 1% GCS dispersion. After the bentonite cake samples were immersed in water, the linear swelling rate increased rapidly. After 16 h, the swelling rate of the bentonite samples in water reached 55.8%. This is because water molecules can enter the clay layers without hindrance of inhibitors, causing significant hydration and swelling of the clay layers. In contrast, the linear swelling rates of the bentonite samples in 5% KCl solution and 1% polyamine solution were 37.4% and 21.5%, respectively. The linear swelling rate of bentonite in 1% GCS decreased significantly to 14.8%, indicating that GCS is more effective than KCl and polyamine in inhibiting the hydration and swelling of clay minerals, which is beneficial for maintaining wellbore stability.

Although all three inhibitors have good inhibitory effects on clay hydration and swelling, the growth patterns of the linear swelling curves are different. The linear swelling of KCl increases rapidly in the first 30 min and then gradually stabilizes. This may be due to the fact that small cations stabilize the clay mineral lattice through ion exchange with the interlayer cations of the clay minerals. Therefore, in the early stage, a large amount of water can enter the interlayer of the clay minerals, causing a rapid increase in the swelling rate. As the ion exchange process ends, the swelling of the clay minerals gradually stabilizes [30]. However, when we observe the results of GCS, the swelling rate of the clay in GCS is higher than that of KCl and polyamine in the early stage. The reason is that KCl and polyamine inhibit the hydration of clay minerals by entering the interlayer of the clay minerals and displacing water molecules in the early stage. However, GCS nanoparticles adsorb on the surface of clay particles, and over time, a dense hydrophobic film gradually forms on the surface of the clay, significantly hindering the invasion of water into the interlayer of the clay crystals.

A 14.8% linear expansion rate (vs. 37.4% for KCl) confirms GCS’s superior chemical inhibition of clay hydration, effectively mitigating shale swelling-induced wellbore collapse.

#### 2.2.3. The Shale Rolling Recovery Test

**Figure 10:**
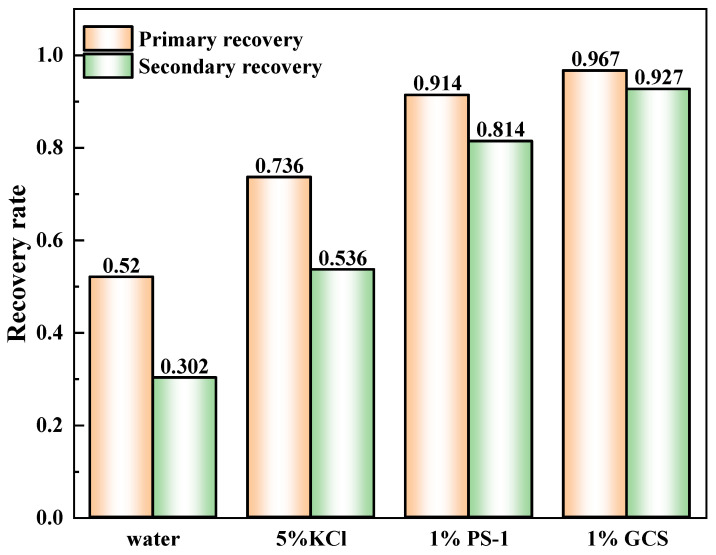
The hot-rolling recovery rate of water, inhibitors and GCS.

The recovery rates of the cuttings after hot rolling in 1 wt% GCS, 1 wt% polyamine, and 5 wt% KCl solutions were further evaluated, as shown in Figure 10. The results indicated that the recovery rate of the shale cuttings in pure water was only 52%. This was because the clay minerals were prone to hydration and dispersion in pure water, leading to a reduced recovery rate. However, in a 5 wt% KCl solution, the recovery rate reached 73.6%, suggesting that small cations have a certain inhibitory effect on clay hydration. Under the inhibition of 1 wt% polyamine, the recovery rate of the cuttings reached 91.4%. Surprisingly, 1% GCS could increase the recovery rate of the cuttings to 96.7%, indicating that GCS has excellent performance in inhibiting clay hydration and dispersion [25,26]. It is crucial for maintaining wellbore stability. Further, the recovery rates of the cuttings after secondary hot rolling in different solutions were determined to evaluate the durability of the chemical agents. The results showed that after secondary hot rolling, the secondary recovery rate of the cuttings treated with GCS only slightly decreased, indicating a strong interaction between GCS and the shale cuttings. Even when the shale was washed and re-exposed to water, the inhibitory effect of GCS still existed, demonstrating long-term inhibition. Compared with KCl and PA, the GCS inhibitor was more effective in inhibiting the hydration and dispersion of shale. For the cationic amine inhibitor (PA), it showed comparable inhibitory performance to GCS at 77 °C, but in the secondary recovery experiment at 150 °C, the inhibitory effect significantly weakened, and the secondary recovery rate of the cuttings was less than 90%. This was due to the hydrolysis of the ether bond in PA at high temperatures. The 96.7% cuttings recovery (exceeding API 90% threshold) validates GCS’s dual action in maintaining shale structural integrity, a critical metric for borehole stability.

### 2.3. Effect of GCS on Drilling Fluid Properties

#### 2.3.1. Effect of GCS on Rheological Properties of Drilling Fluid

Good compatibility is a prerequisite for the application of drilling fluid additives. Although micro-nano plugging agents can effectively block formation pores and reduce fluid loss, it may lead to a decrease in the drilling fluid performance, an increase in flow resistance when they have a significant impact on the rheological properties of the drilling fluid. Therefore, when selecting and applying micro-nano plugging agents, it is necessary to strictly evaluate their impact on the rheological properties and ensure good compatibility with the drilling fluid system.

**Figure 11:**
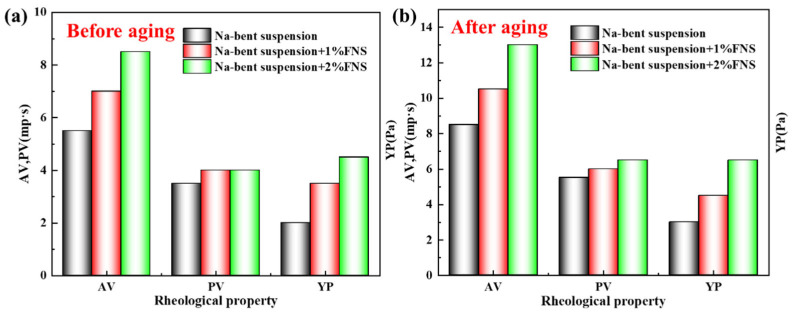
The influence of FNS on the rheological properties of Na-Bent base mud (**a**) Before aging; (**b**) After aging.

Firstly, the influence of FNS on the rheological properties of Na-Bent base mud was investigated, as shown in Figure 11. Apparent viscosity (AV), plastic viscosity (PV), and yield point (YP) are used to describe the rheological characteristics of each experimental mud. It is obvious that different rheological behaviors are exhibited by each system as the concentration of FNS increases. Figure 11 shows the effect of FNS concentration on the rheological properties of the drilling fluid. Before aging, with the increase in FNS concentration, the apparent viscosity, plastic viscosity, and shear force of the drilling fluid all increase slightly. The apparent viscosity of the 4% bentonite slurry is 5.5 mPa·s, and the shear force is 2 Pa. When 1% FNS is added to the bentonite slurry, the apparent viscosity of the base mud increases to 7 mPa·s, and the shear force increases to 3.5 Pa. When the FNS concentration reaches 2%, the apparent viscosity (AV) of the base mud increases from 5.5 mPa·s to 8.5 mPa·s, the plastic viscosity (PV) increases from 3.5 mPa·s to 4 mPa·s, and the shear force increases from 2 Pa to 4.5 Pa. After aging at 150 °C, the viscosity of the aged drilling fluid increases due to the change in the structure and dispersion of bentonite caused by high temperature. The rheological properties of the drilling fluid containing FNS show a similar trend to that before aging. With the increase in FNS concentration, the AV, PV, and dynamic shear force of the drilling fluid all show an increasing trend. Due to its extremely large specific surface area, the nanoparticles can interact strongly with the clay particles and other components in the drilling fluid. Under this strong interaction, they form physical connections between clay particles, constructing a more complex spatial structure, which leads to an increase in apparent viscosity. At the same time, the nanoparticles promote the formation of a three-dimensional network structure, thereby increasing the gel strength in the static state. Overall, the addition of FNS will slightly increase the rheological properties of the Na-bentonite suspension.

**Figure 12:**
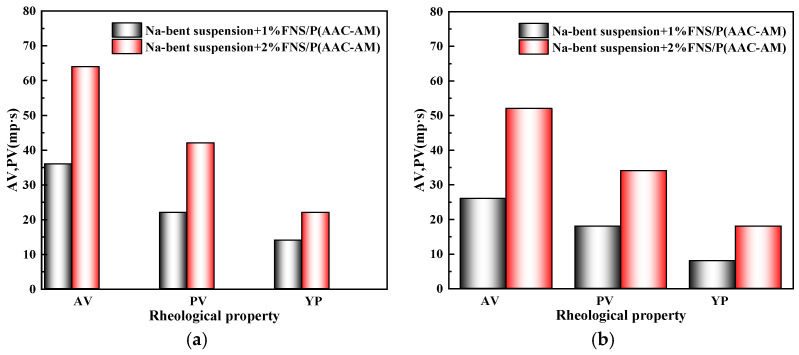
The influence of FNS on the rheological properties of Na-Bent base mud; (**a**) before aging; (**b**) after aging at 150 °C.

Furthermore, after the GCS dynamic gel system was added to the drilling fluid, the viscosity of the system increased sharply, showing in Figure 12. At a 1% dosage, the AV of the drilling fluid before aging increased to 36 mPa·s, the PV increased to 22 mPa·s, and the YP increased to 14 Pa. After aging at 150 °C for 16 h, the AV remained at 26 mPa·s, the PV was 18 mPa·s, and the YP was 8 Pa. At a 2% increase, the AV of the drilling fluid before aging increased to 64 mPa·s, the PV increased to 42 mPa·s, and the YP increased to 22 Pa. After aging at 150 °C for 16 h, the AV remained at 52 mPa·s, the PV was 34 mPa·s, and the YP was 18 Pa. The results show that the addition of GCS has a significant effect on increasing the viscosity of the drilling fluid, indicating the formation of a dynamic gel structure. Notably, YP values (22 Pa unaged; 18 Pa aged) substantially surpassed the API-recommended range (5–15 Pa), indicating exceptional suspension capacity even after thermal degradation. These results demonstrate compliance of GCS with industry standards. All rheological parameters consistently met or exceeded API 13B-1 requirements before and after aging. Further, higher YP retention over 80% at 2% GCS confirms the integrity of the dynamic gel structure under extreme downhole conditions. The 22 Pa yield point (2% dosage) enables efficient hole cleaning at low flow rates, directly reducing stuck pipe risk—a key operational objective for wellbore stability.

#### 2.3.2. The Influence of GCS on Filtration Performance of Na-Bent Base Fluid

**Figure 13:**
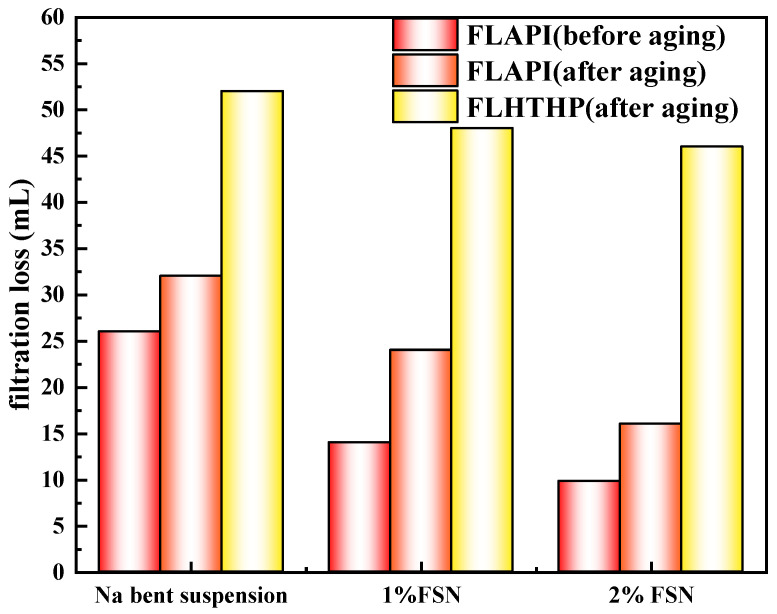
The influence of FSN on filtration performance of Na-Bent base fluid.

**Figure 14:**
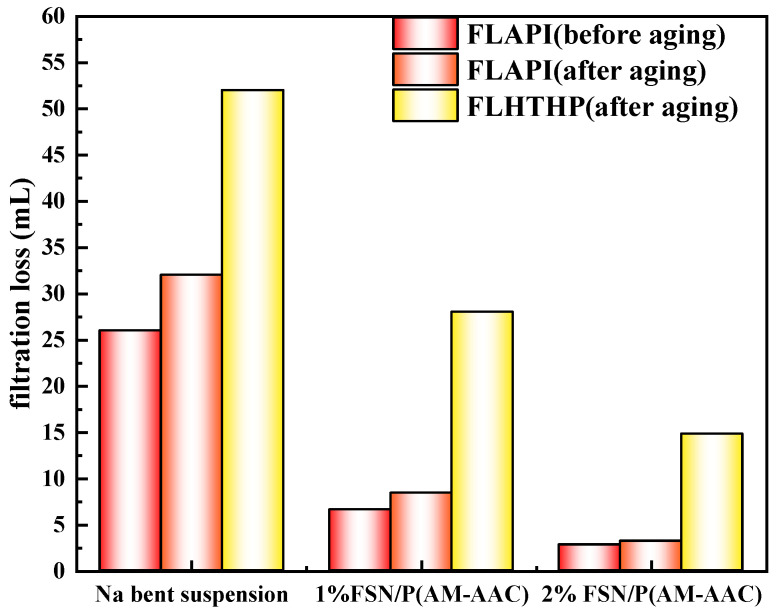
The influence of FSN/P(AM-AAC) on filtration performance of Na-Bent base fluid.

Micro-nano plugging agents can reduce the filtration volume of drilling fluid to a certain extent. We further evaluate the effect of FNS and FNS/P(AM-AAC) on the filtration performance of drilling fluid, as shown Figure 13 and Figure 14. Before aging, when 1% FNS was added, the filtrate loss of drilling fluid decreased from 26 mL without FNS to 14 mL; when the addition amount increased to 2%, the filtrate loss decreased to 9.8 mL. After aging at 150 °C, the filtrate loss of drilling fluid increased. The filtrate loss of drilling fluid without FNS increased to 32 mL, while when 1% FNS was added, the filtrate loss decreased to 24 mL; when the addition amount increased to 2%, the filtrate loss of drilling fluid decreased to 16 mL. The results show that when drilling fluid is affected by high temperature, it will also cause the filter cake to thicken and the permeability to decrease, resulting in an increase in fluid loss. However, FNS can significantly reduce the filtrate loss of drilling fluid to a certain extent. This is mainly because FNS is a nano-scale particle that can effectively fill and embed in the filter cake, block pores, and reduce fluid loss. However, a single FSN particle is insufficient for filtration loss control. The high-temperature and high-pressure filtration loss was further evaluated, and the results are shown in the figure. After being aged at 150 °C for 16 h, the high-temperature and high-pressure filtration loss of 4% bentonite suspension was 52 mL. When 1% FSN was added, the high-temperature and high-pressure filtration loss was reduced to 48 mL. The 2% FSN reduced the high-temperature and high-pressure filtration loss to 46 mL, which is still a relatively large filtration loss and is difficult to meet the drilling requirements in engineering. The results of API and high-temperature and high-pressure fluid loss both indicate that a single nanomaterial can reduce the fluid loss of drilling fluid to a certain extent, but this reduction is difficult to meet the requirements.

We further evaluated the filter loss control effect of the FSN/P(AM-AAC) system. The results show that the dynamic gel system formed by FSN/P(AM-AAC) can significantly reduce the fluid loss of drilling fluid. The 1% FSN/P(AM-AAC) reduced the API loss of the system to 8.4 mL. When the concentration increased to 2%, the API loss of the drilling fluid decreased to 3.2 mL. Under high-temperature conditions, after the drilling fluid was aged at 150 °C, the high-temperature and high-pressure fluid loss of the drilling fluid decreased from 52 mL to 28 mL when the dosage was increased by 1%. As the dosage increased to 2%, the high-temperature and high-pressure fluid loss of the drilling fluid decreased to 14.8 mL. The 52% API filtrate reduction achieved by GCS’s nano-pore sealing and viscosity synergy directly minimizes water invasion into shale, addressing hydration-triggered instability.

### 2.4. Mechanism of FSN/P(AM-AAC)

The effects of electrostatic interaction between FSN and bentonite on shale wettability, capillary force and microstructure were analyzed by zeta potential measurement, contact angle experiment, scanning electron microscope observation and capillary force experiment. Based on the above experimental results, the mechanism of FSN inhibiting shale hydration and stabilizing wellbore walls is proposed.

#### 2.4.1. Micromorphology Analysis

**Figure 15:**
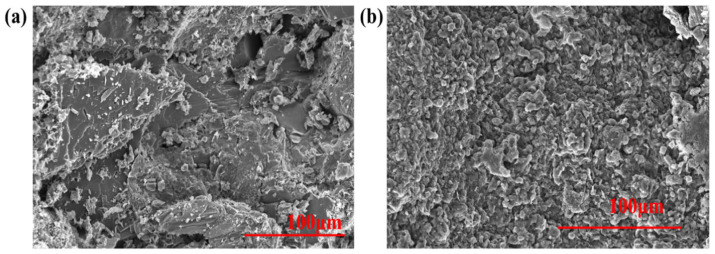
The surface morphology of shale samples treated by (**a**) SiO_2_ and (**b**) FSN.

The cuttings from the shale rolling recovery experiment were collected, and the changes in the microstructure of the cuttings’ surface before and after the GCS treatment were observed by SEM. Figure 15a shows the SEM image of the original shale cuttings, revealing that the surface of the original shale cuttings is rough and has a large number of pores, which provide channels for water to invade the rock, making the rock prone to hydration when exposed to water. Figure 15b shows the microstructure of the cuttings after treatment with 1% GCS dispersion. In contrast, after treatment with 1% GCS, the surface of the cuttings becomes more compact and smooth, and the tiny pores and fissures on the surface are filled with nanoparticles [12,16,18]. The microstructure of the rock surface proves that GSC changes the micromorphology of the rock sample surface through physical adsorption and filling, thereby ensuring that the rock sample is less prone to hydration instability. The gel system can deposit a layer of polymer film-like substances on the rock surface, thereby effectively reducing the intrusion of filtrate into the formation. Nanoparticle penetration into micro fractures visualizes the “physical shield” mechanism, fulfilling the core aim of micro-scale barrier formation against hydration.

#### 2.4.2. Contact Angle

**Figure 16:**
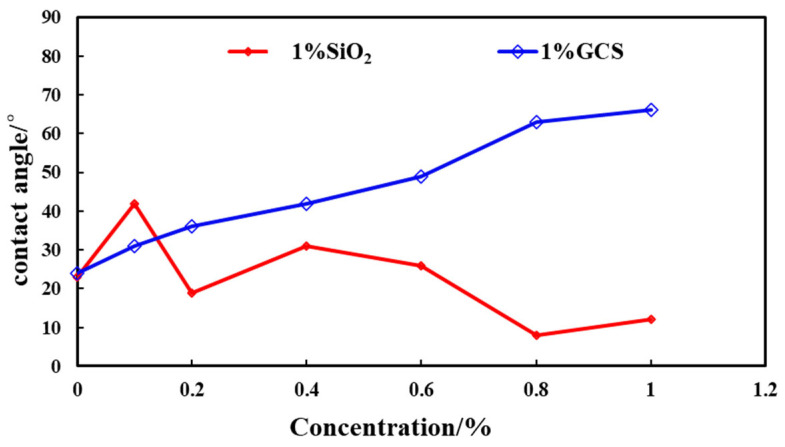
Contact angles of shale samples treated with SiO_2_ and FSN.

The influence of GCS concentration on the contact angle was investigated and compared with that of unmodified nano-SiO_2_. As shown in Figure 16, with the increase in concentration, the contact angle of the solid surface treated with unmodified nano-SiO_2_ gradually decreased, and the sample was highly hydrophilic. This was mainly due to the large specific surface area of the nanoparticles, which increased the contact area between the solid and the liquid after adsorption on the solid surface. The hydrophilic hydroxyl groups on the solid surface increased, and the attraction with water molecules was enhanced, thereby increasing the hydrophilicity. With the increase in GCS concentration, the contact angle of the core sample surface rapidly increased. When the GCS concentration reached 1%, the contact angle exceeded 60°. As the concentration continued to increase, the contact angle of the system gradually stabilized [17,19]. The results indicated that GCS could significantly increase the contact angle and change the wettability from hydrophilic to hydrophobic. The error bars indicated that the experimental error was small and the results were statistically significant. Hydrophobic transformation of shale surfaces proves GCS’s chemical inhibition efficacy, a strategic solution to weaken fluid-shale interactions.

**Figure 17:**
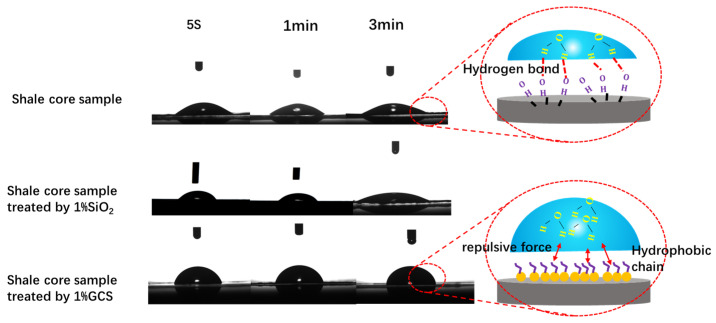
The stability of water contact angles of fresh shale sample and treated by SiO_2_ and FSN.

The dynamic changes in the contact angles of the original shale surface, 1% SiO_2_ treated shale surface and 1% GCS treated shale surface are shown in Figure 17. The water contact angle of the original shale is only 23°, indicating a strong hydrophilic property. After the water droplet is placed on the shale surface, it spreads directly to the surroundings, as shown in Figure 16. This is mainly due to the presence of a large number of hydrophilic hydroxyl groups on the shale surface, which form hydrogen bonds with water molecules and cause the water molecules to spread on the rock surface. The contact angle of the shale surface treated with 1% SiO_2_ further decreases, and the spreading speed is faster. After being soaked in 1% GCS dispersion, the shale surface becomes hydrophobic, with a contact angle of over 60°, and the contact angle remains basically unchanged for a period of time, showing strong stability. This is mainly because FSN nanoparticles are adsorbed on the shale surface, forming a micro–nano rough structure. In addition, the hydrophobic chains on the surface of the nanoparticles face outward, forming a hydrophobic repulsive force with water molecules, hindering the adsorption and spreading of water molecules. The existence of the micro–nano rough structure and hydrophobic chains meets the conditions for constructing a hydrophobic interface, thereby transforming the wettability of the shale surface into a closed hydrophobic surface.

#### 2.4.3. The Capillary Force Test

Micro-pores and fractures are widely developed in shale formations. The capillary action is particularly significant when drilling in hydrophilic shale reservoirs. Driven by capillary forces, water is sucked into the shale, causing it to destabilize due to hydration. In order to study the effect of GCS on the capillary force of shale, the glass capillary rise experiment was carried out.

**Figure 18:**
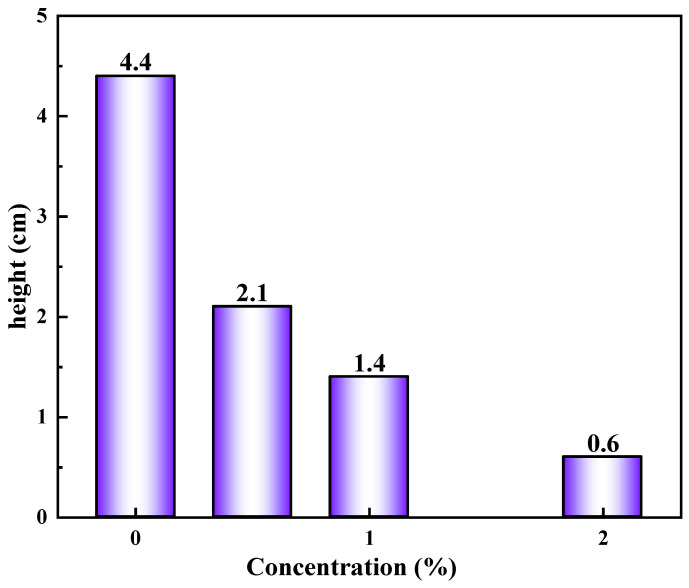
The rising height of the water in the glass capillary tube after soaking with different concentrations of FSN.

Figure 18 shows the rising height of the water in the glass capillary tube after soaking with different concentrations of GCS. The experimental results show that the rising height of water in the untreated capillary is 4.4 cm, which indicates that the surface of the capillary has strong hydrophilicity, and the capillary force is large, which can significantly drive water molecules into the capillary. However, after different concentrations of GCS (hydrophobic surfactant modified nano-silica particles) were soaked, the rise height of water decreased significantly, and with the increase in GCS concentration, the rise height further decreased. In the capillary treated with 0.5% GCS, the water rise height was 2.1 cm. When the concentration of GCS is 1%, the rising height of water is 1.4 cm. When the concentration of GCS reaches 2%, the rising height of water decreases to only 0.6 cm. The size of capillary force is closely related to the wettability of capillary surface. Untreated capillary surface is highly hydrophilic, and the interaction between water molecules and the inner wall of the capillary is large, so water can rise quickly and reach a higher height. However, the surface of the capillary treated with GCS was covered by hydrophobic silica nanoparticles, and the surface wettability changed from hydrophilic to closed hydrophobic, and the interaction between water molecules and the inner wall of the capillary was weakened, resulting in a decrease in the capillary force and a decrease in the rise height of water. In the process of drilling, shale formation has smaller pores and strong hydrophilicity, resulting in large formation capillary force. Strong capillary forces will spontaneously suck fluid into the formation, causing wellbore instability. GCS can significantly reduce capillary force and inhibit fluid intrusion into formation pores.

#### 2.4.4. Mechanism Analysis

(1) Physical blocking effect of nanoparticles

The modified nano-silica FSN has an extremely small particle size and can effectively fill the pores and micro-fractures of shale in drilling fluid. These nanoparticles, with their tiny size and good dispersibility, can enter and closely pack in the pores and fractures, forming a physical barrier that prevents the further invasion of drilling fluid into the shale, thereby reducing the filtrate loss of drilling fluid. At the same time, this physical blocking effect enhances the integrity of the wellbore surface, prevents excessive contact between shale and drilling fluid, and inhibits the dispersion of shale due to hydration, ultimately improving the inhibition performance of shale hydration and dispersion and enhancing the stability of the wellbore.

(2) Reducing capillary force

When drilling fluid comes into contact with shale, the capillary force existing in the pores of shale will cause the filtrate of drilling fluid to penetrate into the interior of shale. After the modified nano-silica FSN is added to the drilling fluid, it can adsorb on the surface of shale pores and change the surface properties of the pores. Its special hydrophobic structure reduces the hydrophilicity of the pore surface, thereby reducing the capillary force between the drilling fluid and the shale pores. This reduction in capillary force effectively hinders the invasion of drilling fluid filtrate under capillary action, thereby reducing the filtrate loss of drilling fluid. At the same time, it also reduces the risk of shale hydration and dispersion due to excessive absorption of filtrate, enhancing the stability of the wellbore. At the same time, the change in the surface properties of shale inhibits the interaction between shale and water molecules, improving the inhibition performance of shale hydration and dispersion, which is beneficial to maintaining the stability of the wellbore.

(3) Forming dynamic weak gel structure

**Figure 19:**
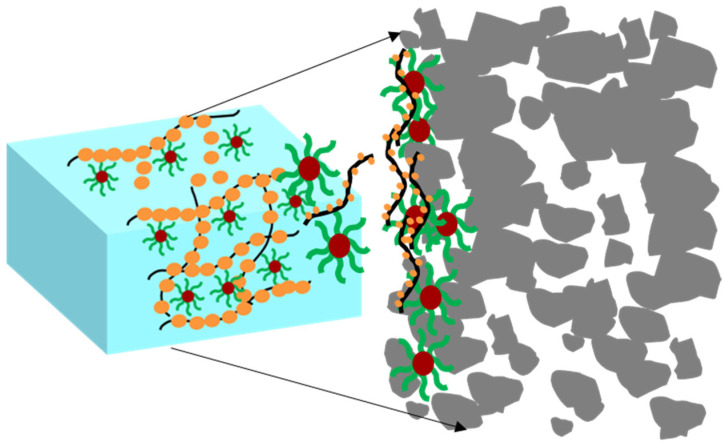
Schematic diagram of the mechanism by which dynamic cross-linked weak gel improves the rheological and fluid loss performance of drilling fluid.

Figure 19 demonstrates a schematic diagram of the mechanism by which dynamic cross-linked weak gel improves the rheological and fluid loss performance of drilling fluid. The synergistic interaction between KH550-modified nano-silica and the copolymer P(AM-AAC) (polyacrylamide–acrylic acid) establishes a 3D dynamic hydrogen-bonded network, which significantly enhances the rheological properties of drilling fluids. Nano-silica particles are grafted with the silane coupling agent KH550, introducing primary amine groups (-NH_2_) on their surfaces. These amine groups act as hydrogen-bond donors. The carboxyl groups (-COOH) and amide groups (-CONH_2_) in P(AM-AAC) serve as hydrogen-bond acceptors. The KH550-functionalized nano-silica and P(AM-AAC) copolymer collaboratively construct a shear-thinning dynamic hydrogel via reversible hydrogen bonding. The amine groups (-NH_2_) on silica surfaces form multiple H-bonds with carboxyl (-COOH) and amide (-CONH_2_) moieties in P(AM-AAC), creating a hybrid 3D network. This gel significantly elevates the apparent viscosity of the drilling fluid, immobilizing free water through hydrodynamic confinement and capillary entrapment. Concurrently, nano-silica particles seal formation pores, synergistically reducing filtrate invasion. The dynamic nature of H-bonding further imparts self-adaptive rheology, ensuring pumpability under shear while maintaining filtration control at static conditions.

## 3. Conclusions

This study successfully developed a dynamic hydrogen bond cross-linked P(AM-AAC)/SiO_2_@KH550 nanocomposite hydrogel (GCS) by integrating hydrophobically modified nano-SiO_2_ (FSN) with acrylamide–acrylic acid copolymer. The design directly addresses the core challenge of wellbore instability, as the primary barrier to efficient and safe drilling operations. Experimental validation confirms GCS’s superiority:

(1) Hydration inhibition: 1% GCS reduces linear expansion to 14.8% (vs. KCl: 38.2%; polyamine: 25.7%) and increases cuttings recovery to 96.7% (vs. KCl: 72%; polyamine: 85%)—exceeding API standards for recoverable shale integrity (>90%) 27.
(2) Rheology-filtration balance: The gel network elevates drilling fluid viscosity (YP: 22 Pa at 2% dosage) while reducing API filtrate loss by 52% through dual pathways:
(3) Thermal endurance: After aging (150 °C/16 h) YP retention ≥ 82% validates field applicability in deep reservoirs.

The innovation of GCS lies in two main transformative advancements over current inhibitors. Firstly, hydrophobic nano-SiO_2_ (penetrating micro-fractures) and dynamic polymer networks (forming hydrogen-bonded barriers) are creatively combined to achieve simultaneous physical plugging and chemical inhibition—overcoming the singular-action limitations of conventional inhibitors like KCl (ionic suppression only) or polyamine (surface adsorption only). Additionally, the hydrogen-bonded network exhibits shear-thinning viscosity (unlike rigid structures in polyurethane pluggers), enabling adaptive sealing under downhole stress fluctuations.

In summary, GCS redefines wellbore stabilization through molecular-scale engineering, offering a field-ready solution that bridges the gap between nanomaterial potential and operational reality.

## 4. Materials and Methods

**Figure 20:**
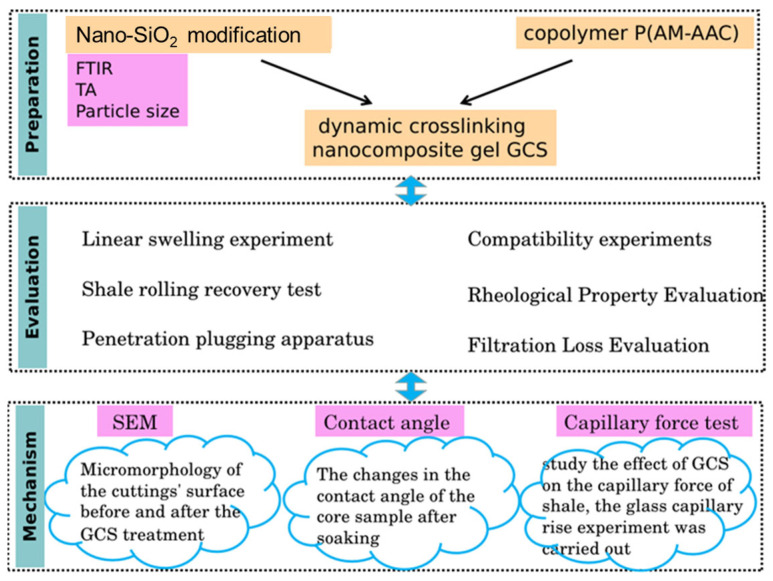
A flow chart demonstrating the methodology.

Figure 20 shows a flow chart demonstrating the methodology of our work. Firstly, modified nano-silica and dynamic cross-linked gel were prepared, and the molecular structure of the synthesized product were characterized by experimental means. Next, the sealing performance, the performance of inhibiting the hydration of mud shale, as well as the rheological and fluid loss performance of the drilling fluid were evaluated. Finally, the application mechanism of this dynamic cross-linked gel material in drilling fluid was studied by various means.

### 4.1. Materials

Nano-SiO_2_ (Particle size 15–20 nm) was purchased from Macklin Biochemical TecFSNology Co., Ltd. (Shanghai, China), and NaOH was purchased from Shanghai Aladdin Biochemical TecFSNology Co., Ltd. (Shanghai, China). AM and AAC were also purchased from Shanghai Aladdin Biochemical TecFSNology Co., Ltd. (Shanghai, China). The Calcium bentonite (Ca-Bent) used for the linear swelling test and sodium bentonite (Na-Bent) used for drilling fluid preparation were purchased from Shandong Surabaya Saint Feng Bentonite Co., Ltd. (Jining, China). Shale samples used in this paper were obtained from southwest oil filed in China.

### 4.2. Preparation of GCS

SiO_2_ was activated by drying at 40 °C for 48 h to ensure the activation of the required functional groups. Then, the dried SiO_2_ was mixed with deionized water and anhydrous ethanol in a flask and ultrasonically stirred for 2 h to achieve good dispersion. Subsequently, KH550 (3 wt%) was added and stirred for 30 min. After the system was uniformly mixed, the reactor was heated in a water bath at 80–90 °C for 4 h to promote the desired chemical interaction between SiO_2_ and KH550. Finally, the nano-powder was washed with anhydrous ethanol three times and then dried at 100 °C for 3 h to obtain the sample, which was labeled as FSN, SiO_2_@KH550.

P(AM-AAC) was polymerized with the prepared SiO_2_@KH550 as a dynamic cross-linking agent to obtain the physically cross-linked nanocomposite hydrogel P(AM-AAC)/SiO_2_@KH550. The typical preparation process is: Dissolve 0.05 g of SiO_2_@KH550 mixed nanoparticles in 50 mL of deionized water and ultrasonically disperse to obtain a uniform solution. Then, 15 g of AM and AAC were added to the above solution. Finally, in a nitrogen atmosphere, the initiator APS was added, and the polymerization reaction was carried out at 70 °C for 6 h. The hydrogel obtained was denoted as GCS, (P(AM-AAC)/SiO_2_@KH550.

### 4.3. Structural Characterizations of GCS

The chemical structure, thermal stability, electrification and microstructure of GCS were analyzed by Fourier infrared spectroscopy (FT-IR), thermogravimetric analysis (TGA), potential analysis, scanning electron microscopy (SEM) and transmission electron microscopy (TEM).

(1) FT-IR

Infrared spectrometers are used to analyze the molecular structure and chemical composition of substances absorbing infrared radiation of different wavelengths. Fourier infrared spectroscopy was utilized to determine the molecular structure of the prepared FSN. The FT-IR was obtained by the KBr press method with a scanning range of 400–4000 cm^−1^ with a resolution of 4 cm^−1^ on a Nicolet 6700 FT-IR Spectrometer (Thermo Fisher Scientific Corporation, Waltham, MA, USA).

(2) Thermogravimetric analyze

The thermal stability of unmodified nano-SiO_2_ and FSN were evaluated using a Dupont 2100 thermogravimetric analyzer. The experimental temperature range was 40 °C to 800 °C, with a heating rate of 10 °C·min^−1^.

(3) Particle size

Unmodified and nano-SiO_2_ and FSN were dispersed in deionized water and subjected to ultrasonication for 10 min to ensure thorough dispersion of the particles. The particle size distribution of the resulting nano-dispersions was measured using NanoBrook Omni apparatus (Nashua, NH, USA) based on Dynamic Light Scattering (DLS). Each sample was measured in triplicate to ensure the reproducibility and reliability of the data. Additionally, the particle size distribution of FSN dispersion was evaluated before and after aging at 150 °C to assess the impact of high-temperature exposure on the stability of FSN [26,27].

(4) Microscopic morphology observation

Scanning electron microscopy (SEM) and transmission electron microscopy (TEM) were used to observe the morphology of nano-SiO_2_ before and after modification. TEM was conducted on the JEM 2100F transmission electron microscope (Nippon Electronics Co., Ltd., Tokyo, Japan). The morphology of nanoparticles before and after modification was detected by filed scanning electron microscopy (HITACHI UHR FE-SEM SU8010, Hitachi, Japan) [18,21].

### 4.4. Method of Evaluation

Linear expansion experiment, shale rolling recovery experiment and spontaneous imbibition experiment were used to evaluate the anti-collapsing effect of FSN, and the influence of FSN on the rheological properties and filtration properties of the base pulp was also evaluated.

#### 4.4.1. Linear Expansion Experiment

Linear swelling experiment can be used to characterize the hydration degree of shale. Shale will hydrate and expand after contact with water-based drilling fluid, and this expansion behavior will significantly affect the stability of borehole walls. Through linear expansion experiment, the size change in shale during hydration can be quantitatively analyzed, so as to evaluate its hydration degree.

NP01expansion meter tester was employed from Qingdao Chuangmeng Instrument Technology Service Co., Ltd. (Qingdao, China). The inhibition effect of linear swelling was evaluated by selecting Ca-Bent which expanded rapidly in water in a relatively short time. The dried Ca-Bent powder (10 g) was compacted at 10 MPa for 5 min using a hydraulic press and a cylindrical mold to obtain a bentonite sample (about 25 mm in diameter and 10 mm in height). The prepared Ca-Bent sample is placed in a linear dilatometer with the initial expansion height set to zero, and then different test solutions are added. The variation in expansion height over time were recorded [27,28].

#### 4.4.2. The Shale Rolling Recovery Test

The shale rolling recovery test is an important method to evaluate the ability of drilling fluid to inhibit the hydration and dispersion of shale. By simulating the thermal stability and inhibition effect of mud shale in drilling fluid, the experiment can directly reflect the inhibition performance of drilling fluid and treatment agent on mud shale. Weigh 30.0 g shale cuttings with the size of 6–10 mesh and 210 mL drilling fluid, pour into the aging tank, and heat roll at high temperature for 16 h. After cooling to room temperature, the rock cuttings were screened and washed for 1 min with a sample sieve with a hole size of 40 mesh, and then the cuttings were dried in a constant-temperature drying oven at 105 ± 3 °C for 4 h, and then the remaining shale cuttings were weighed, marked as W1. Finally, the rolling recovery rate of shale was obtained by calculation. The recovered cuttings were put back into the aging tank, 210 mL of water was added, and hot rolled again for 8 h. The cuttings were recovered in the same way, and the mass W2 was recorded. The recovery rate is determined by the following formula: primary recovery rate (FRR) = W1/30 × 100%, and secondary recovery rate (SRR) = W2/30 × 100%.

#### 4.4.3. Penetration Plugging Apparatus

The Fann penetration plugging apparatus (PPA) simulates and measures static downhole fluid loss. The 5000 psi PPA accurately predicts how the drilling fluid will form a permeable cake to plug losses in the presence of differential pressure. The permeability sealing tester simulates formation temperatures using a conventional high-temperature and high-pressure heating jacket. The mud cup is placed in reverse, the pressure enters from the bottom of the mud cup, and the filtrate is collected from the top. A small manual hydraulic pump is used to pressurize the mud cup and pressure enters the drilling fluid sample. PPA tester uses ceramic filters, which are available in a variety of porosity options.

#### 4.4.4. Compatibility Experiments

To evaluate the compatibility of GCS with drilling fluids, the effects of GCS concentration on the rheological and filtration properties of drilling fluids need to be systematically assessed. The specific methodology and procedures are as follows:

(1) Rheological Property Evaluation: Utilize a 6-speed rotational viscometer to measure the apparent viscosity, plastic viscosity, and yield point of each drilling fluid sample at different shear rates and temperatures. This step is crucial for understanding how FSN affects the fluid’s ability to suspend cuttings and maintain stability during drilling.
(2) Filtration Loss Evaluation: Use a standard API filtration cell to measure the filtrate loss of each drilling fluid sample under 0.7 MPa. Filtrate loss is a critical parameter for assessing the ability of the drilling fluid to form an effective filter cake and minimize fluid invasion into the formation.

### 4.5. Mechanism Analysis

#### 4.5.1. SEM

The cuttings from the shale rolling recovery experiment were collected, and the changes in the microstructure of the cuttings’ surface before and after the GCS treatment were observed by SEM [31].

#### 4.5.2. Contact Angle

Contact angles were tested using JC2000D5M Contact Angle tester from Shanghai Zhongchen Digital Technology Equipment Co., Ltd. (Shanghai, China). The shale core pieces are cut to 25 mm in diameter and 0.5 cm in thickness. The 1200-mesh sandpaper shale sheet was used for polishing to avoid the influence of the heterogeneity of the core sample on the experimental repeatability. The prepared shale samples were put into the aging tank, soaked in 1% FSN dispersion, and then the container was sealed and heated in the incubator at 150 °C for 16 h. After that, the shale sheet is removed and dried, and the contact angle is determined using a contact angle meter. Each sample was tested three times, and the mean and error bars were calculated [17,18,30,31].

#### 4.5.3. The Capillary Force Test

A capillary test is an experimental method used to measure capillary force or the height of capillary water rise, and is often used to study the wettability of materials, pore structure, and the behavior of liquids in tiny channels. Specific experimental steps are as follows [18,30]:

① Clean the capillary tube with alcohol cotton to remove grease or impurities.
② Soak the cleaned capillaries in different concentrations of FSN liquid, fully treat them for 24 h, and then dry the capillaries in the oven again to ensure that there is no residual liquid in the tube.
③ Fix the capillary vertically on the support frame with clamps to ensure that the lower end is not tilted when immersed in liquid. Fill the beaker with an appropriate amount of liquid (such as pure water) and slowly insert it vertically into the capillary tube to avoid bubbles.
④ Measure the height of the liquid column: after the liquid level is stable, use a scale to measure the height difference between the liquid level outside the tube and the liquid level inside the capillary. Repeat the average 3 times to reduce the error.

## Data Availability

The original contributions presented in this study are included in the article. Further inquiries can be directed to the corresponding author.
